# SUN5 Interacting With Nesprin3 Plays an Essential Role in Sperm Head-to-Tail Linkage: Research on Sun5 Gene Knockout Mice

**DOI:** 10.3389/fcell.2021.684826

**Published:** 2021-06-29

**Authors:** Yunfei Zhang, Linfei Yang, Lihua Huang, Gang Liu, Xinmin Nie, Xinxing Zhang, Xiaowei Xing

**Affiliations:** ^1^Center for Experimental Medicine, The Third Xiangya Hospital, Central South University, Changsha, China; ^2^Department of Laboratory Medicine, The Third Xiangya Hospital, Central South University, Changsha, China; ^3^The Institute of Reproduction and Stem Cell Engineering, Central South University, Changsha, China

**Keywords:** LINC complex, SUN5, Nesprin3, centrosome, sperm head-to-tail linkage

## Abstract

Acephalic spermatozoa syndrome is a rare genetic and reproductive disease. Recent studies have shown that approximately 33–47% of patients with acephalic spermatozoa syndrome have SUN5 mutations, but the molecular mechanism underlying this phenomenon has not been elucidated. In this study, we generated Sun5 knockout mice and found that the head-to-tail linkage was broken in Sun5^–/–^ mice, which was similar to human acephalic spermatozoa syndrome. Furthermore, ultrastructural imaging revealed that the head-tail coupling apparatus (HTCA) and the centrosome were distant from the nucleus at steps 9–10 during spermatid elongation. With the manchette disappearing at steps 13–14, the head and the tail segregated. To explore the molecular mechanism underlying this process, bioinformatic analysis was performed and showed that Sun5 may interact with Nesprin3. Further coimmunoprecipitation (Co-IP) and immunofluorescence assays confirmed that Sun5 and Nesprin3 were indeed bona fide interaction partners that formed the linker of the nucleoskeleton and cytoskeleton (LINC) complex participating in the connection of the head and tail of spermatozoa. Nesprin3 was located posterior and anterior to the nucleus during spermiogenesis in wild-type mice, whereas it lost its localization at the implantation fossa of the posterior region in Sun5^–/–^ mice. Without correct localization of Nesprin3 at the nuclear membrane, the centrosome, which is the originator of the flagellum, was distant from the nucleus, which led to the separation of the head and tail. In addition, isobaric tag for relative and absolute quantitation results showed that 47 proteins were upregulated, and 56 proteins were downregulated, in the testis in Sun5^–/–^ mice, and the downregulation of spermatogenesis-related proteins (Odf1 and Odf2) may also contribute to the damage to the spermatozoa head-to-tail linkage. Our findings suggested that Sun5 is essential for the localization of Nesprin3 at the posterior nuclear membrane, which plays an essential role in the sperm head-tail connection.

## Introduction

Intact spermatozoids are a critical factor for species reproduction. Before swimming out of the testis, spermatozoa undergo a series of complex and precisely coordinated processes involving acrosome formation, nuclear condensation and elongation, cytoplasm elimination, and flagellum development (Hao et al., 2019). The head-tail coupling apparatus (HTCA) (also called the connecting piece or neck), which is a centrosome-based structure, mediates the tight junction of the sperm head and flagellum. HTCA develops from the centrosome and is composed of proximal centrioles, distal centrioles and pericentriolar materials, including segmented columns and capitulum (Galletta et al., 2020; Wu et al., 2020). The HTCA attaching to the nuclear membrane is a prerequisite for the tight connection between the sperm head and tail, causing decapitation of sperm and infertility when faulty (Galletta et al., 2020; Wu et al., 2020).

Current researchers have found that many proteins, such as Spata6 (Yuan et al., 2015), Hook1 (Mendoza-Lujambio et al., 2002), Oaz-t (Tokuhiro et al., 2009), Cntrob (Liska et al., 2009), Prss21 (Netzel-Arnett et al., 2009), Odf1 (Yang et al., 2012; Yang et al., 2014), FAM46C (Zheng et al., 2019), Spatc1l (Kim et al., 2018), Pmfbp1 (Zhu et al., 2018; Sha et al., 2019), and Sun5 (Shang et al., 2017) are involved in the connection of the sperm head and tail. Spata6 is thought to be a part of the segmented columns and the capitulum, and the knockout of Spata6 disrupts the correct formation of segments in HTCA (Yuan et al., 2015). Odf1, the main component of outer dense fibers, contributes to the head-to-tail linkage and the development of the flagellum. The knockout of Odf1 in mice resulted in decapitated sperm, which is similar to that in humans with acephalic spermatozoa syndrome (Perotti and Gioria, 1981). Yang et al. (2012, 2014) found that the downregulation of Odf1 in mice showed an enlargement of the spacing between the nucleus and capitulum, indicating a weakening of head and tail coupling. Currently, SUN5 (Zhu et al., 2016; Shang et al., 2017; Sha et al., 2018b; Liu et al., 2020), PMFBP1 (Zhu et al., 2018; Sha et al., 2019), BRDT (Li et al., 2017), TSGA10 (Sha et al., 2018a; Liu et al., 2020), and CEP112 (Sha et al., 2020) gene mutations have been reported in acephalic spermatozoa syndrome patients. Among them, the genetic contribution rate caused by SUN5 is approximately 33–47% (Zhu et al., 2016; Sha et al., 2018b), which is the highest among the mutations detected thus far.

SUN5 (also termed SPAG4L) was first cloned and submitted to GenBank by our group and was originally named TSARG4 in humans (GenBank accession AF401350) and SRG4 in mice (GenBank accession AY307077). In our previous studies, we found that Sun5 was specifically expressed in mouse testis, strictly regulated by growth, and highly expressed after postnatal 3 weeks (Xing et al., 2004). Sun5 is detected in all stages from spermatocytes to mature spermatozoa (Xing et al., 2004; Li et al., 2019). In mature spermatozoa, SUN5 is located at the nuclear posterior pole and participates in the junction of the head and tail of spermatozoa (Yassine et al., 2015; Shang et al., 2017).

As the fifth member of the SUN domain, SUN5 contains the conserved SUN domain at the C-terminus, the coiled-coil (CC) domain and the transmembrane (TM) region at the N-terminus. Our previous study showed that the CC and TM domains were essential for SUN5 protein localization to the nuclear envelope (Jiang et al., 2011). Classical SUN proteins (SUN1 and SUN2) can assemble the LINC complex with at least four KASH (Klarsicht, ANC-1, and Syne homology) proteins (Syne/Nesprin-1 to 4), which connect the nucleus to the cytoskeleton (Rothballer et al., 2013; Xu et al., 2018). We found earlier that SUN5 interacts with Nesprin2 to form LINC complexes, playing an important role in the meiotic process (Li et al., 2019).

Nesprin3, also termed Syne3, is characterized by the presence of a C-terminal KASH domain and a spectrin repeat (SR) region. In contrast to Nesprin1 and 2, Nesprin3 cannot link to the cytoskeleton directly due to the lack of an actin-binding domain (ABD) at the N-terminus (Supplementary Figure 1) but can interact with proteins containing ABD domains, such as plectin, BPAG1, and MACF, to indirectly bind to the cytoskeleton (Ketema and Sonnenberg, 2011; Liao et al., 2019). Morgan et al. (2011) reported that Nesprin3 played a crucial role in perinuclear cytoskeletal organization and attachment of the centrosome to the nuclear envelope. The knockdown of Nesprin3 increased the spacing between the nucleus and the centrosome (Morgan et al., 2011). Nesprin3 can form spermiogenesis-specific LINC complexes with Sun1η, a new spermiogenesis-specific Sun1 isoform, anterior to the nucleus and may be involved in acrosomal composition (Göb et al., 2010). Nesprin3 is polarized to the posterior pole in elongated spermatid (Göb et al., 2010), but its function is not clear. Mice with Sun5 knockout showed decapitated sperm with unattached HTCA at the nucleus (Shang et al., 2017), but the molecular mechanism remains elusive. Bioinformatics analysis revealed that Sun5 may interact with Nesprin3; thus, we speculated that Sun5 may cooperate with Nesprin3 to form the LINC complex, playing a significant role in sperm head-to-tail linkage.

In this study, we generated Sun5^–/–^ mice and found that Sun5 deletion caused decapitated sperm in the epididymis, resulting in the phenotype of acephalic spermatozoa and male infertility. Studies on the finer structure of spermatozoa development and the interaction between SUN5 and Nesprin3 explained the mechanism underlying the abnormal expression of SUN5 causing acephalic spermatozoa syndrome, providing new insights into the sperm head-to-tail linkage.

## Materials and Methods

### The Generation and Identification of Sun5^–/–^ Mice

Using TALEN technology, Sun5^–/–^ mice were generated in cooperation with Wuhan Kangweida Company, and the target of gene knockout was exon 4. The mice were kept in a room with specific pathogen-free conditions, controlled light (14:10-h light/dark cycle) and temperature (23 ± 0.5°C). Operations were performed according to laboratory animal management practices and approved by the Experimental Animals Ethics Committee of the Third Xiangya Hospital, Central South University. Genomic DNA was isolated from tail tip biopsy specimens, and genotyping was performed by PCR using Sun5-specific primers (Supplementary Table 1). PCR conditions were as follows: preincubation at 95°C for 2 min, amplification for 35 cycles in three steps (94°C for 30 s, 57°C for 30 s, 72°C for 30 s), and extension at 72°C for 7 min. Then, the PCR products were sequenced, and the results were compared with that of wild-type (WT) mice in NCBI^1^.

### Western Blotting

Testicular tissues collected from WT and Sun5^–/–^ mice were lysed in RIPA buffer supplemented with protease inhibitor. The supernatants collected were separated in 10% SDS-PAGE gels (KeyGEN BioTECH) and transferred onto polyvinylidene fluoride (PVDF) membranes (Millipore). The membrane was blocked with 5% non-fat milk followed by incubation at 4°C overnight using the following antibodies: rabbit Sun5 polyclonal antibody (1:1000, Proteintech), rabbit polyclonal to Nesprin3 (1:1000, Abcam), or mouse GAPDH monoclonal antibody (1:1000, Proteintech). After three washes (5 min each) with TBST (1 × TBS containing 0.1% Tween-20), the membranes were incubated with secondary antibodies conjugated with horseradish peroxidase (1:1000, Proteintech) for 1 h, followed by washing. An enhanced chemiluminescence kit (Proteintech) was used to visualize the protein bands with a UVP ChemStudio PLUS multifunctional imager (UVP).

### Hematoxylin-Eosin Staining

Testes and epididymis samples obtained from 8-week WT and Sun5^–/–^ mice were fixed with 4% paraformaldehyde. After dehydration, paraffin embedding and sectioning, hematoxylin and eosin staining were performed as previously described (Shang et al., 2017). Finally, the sections were observed and photographed under an IX71 microscope (Olympus).

### Transmission Electron Microscopy

Fresh mouse testicular and epididymal tissues were fixed overnight in electron microscopy solution. The samples were dehydrated in an ethanol-acetone gradient and embedded in epoxy resin, and the slice thickness was 60–70 nm. Finally, dioxane acetate and lead citrate were used for staining. Images were taken with a Tecnai G2 Spirit TWIN transmission electron microscope (FEI-TEM).

### Co-immunoprecipitation Assay

We purchased the pLVX-IRES-Puro-SUN5-Flag lentivirus from Yingrun Biotechnologies Inc., and the amount of virus was greater than 10^8^ TU. HepG2 cells were transfected with pLVX-IRES-Puro-SUN5-Flag lentivirus and screened according to the method provided by the company. Stably transfected cells, or testicular tissues milled with liquid nitrogen, were lysed with 1 mL of IP lysis solution (Beyotime) supplemented with cocktail of phosphatase inhibitors (TargetMol). After centrifugation at 12,000 rpm at 4°C for 25 min, the supernatant was removed and divided into 400 μL aliquots. Each sample aliquot contained 8 μg of target antibodies and 8 μg of mock antibodies (IgG) and was incubated with rotation at 4°C overnight. Then, 25 μL of Protein A/G-coated magnetic beads was added to bind the antibody complexes at 4°C for 6 h. After washing with RIPA three times and performing acid elution, the eluents were neutralized and collected for subsequent western blot analysis. Western blot analysis was performed as described above.

### Immunofluorescence Assay

Immunofluorescence of mouse testicular cell smears and spermatozoa smears was performed as we previously described (Li et al., 2019). Briefly, fresh testicular tissue removed from the albuginea and adipose tissue was sheared into pieces in PBS and filtered with a 200-mesh sieve. After washing with PBS three times, performing hypotonic treatment for 15 min, and fixing with 4% paraformaldehyde for 10 min, the cells were smeared on lysine-coated glass slides and air-dried. Subsequently, through 0.5% Triton X-100 permeabilization and 5% BSA blocking, the glass slides were hybridized with the following primary antibodies overnight at 4°C: rabbit Sun5 polyclonal antibody (1:100, Proteintech), rabbit Nesprin3 polyclonal antibody (1:100, Proteintech), and mouse monoclonal to Nesprin3 (1:100, Abcam). After three washes, hybridization with the corresponding fluorescent secondary antibodies, DAPI restaining and glycerol sealing, the fluorescence-stained cells were observed under an IX71 fluorescence microscope (Olympus).

### Isobaric Tag for Relative and Absolute Quantitation Quantification Proteomics Analysis

Testicular proteins extracted from two WT and two Sun5^–/–^ mice at 8 weeks postnatal were isolated and enzymolyzed. Isobaric tag for relative and absolute quantitation (iTRAQ) labeling, sample mixing, high-performance liquid chromatography (HPLC) separation and liquid chromatography-tandem mass spectrometry (LC-MS/MS) analysis were performed after quality control. The raw data files produced by mass spectrometry (MS) and protein quantification were interpreted using Proteome Discoverer software. The raw MS/MS data were converted by the corresponding tool and searched using Mascot version 2.3.02 in this project against the selected database.

### RNA Extraction and Real-Time Quantitative PCR Analyses

Total RNA was extracted from samples using TRIzol Reagent (Invitrogen). The first cDNA strand was synthesized according to the protocol for ReverTra Ace qPCR RT Master Mix with gDNA Remover (TOYOBO). Quantitative PCR (qPCR) was performed with TB Green Premix Ex *Taq*^TM^II (Takara) using specific primers (Supplementary Table 1) in a qPCR machine (Roche LightCycler 480). Gapdh was used as an internal control.

## Data Analysis

Statistical analyses were conducted by GraphPad PRISM version 8, and the statistical significance of different groups was measured by Student’s *t*-test. Blast2GO software was used to analyze Gene Ontology (GO) annotation, and all identified proteins were annotated to three categories: cellular component, molecular function and biological process (*P* < 0.05). The STRING database^2^ was used to construct the protein-protein interaction networks. The DisGeNET database^3^ and Cytoscape software were used to acquire the relationships between differentially expressed proteins (DEPs) and human reproductive diseases.

## Results

### The Generation of Sun5^–/–^ Mice

**FIGURE 1:**
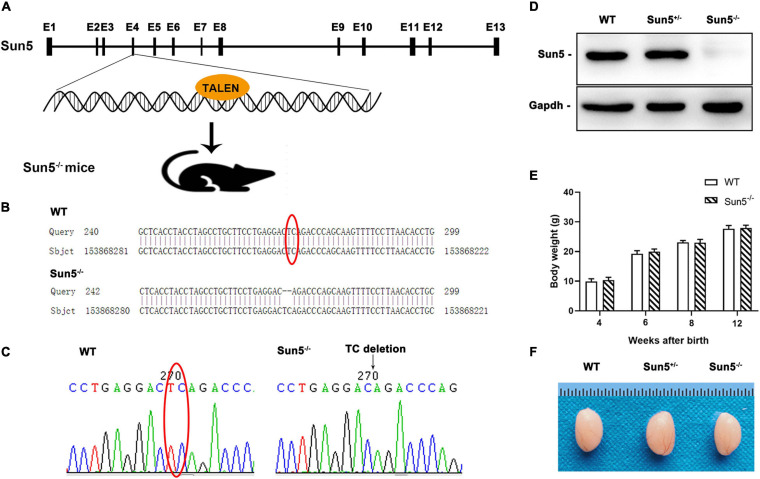
Generation of Sun5^–/–^ mice. **(A)** Schematic illustration of the Sun5^–/–^ mouse knockout strategy. **(B,C)** Sequences of the knockout alleles in Sun5^–/–^ mice determined by Sanger sequencing. **(D)** Western blot results showing the absence of Sun5 proteins in the testes of Sun5^–/–^ mice but detection in WT and Sun5^+/–^ mice. Gapdh served as a loading control. **(E)** Similar body weights of WT and Sun5^–/–^ mice during the growth process. Data are presented as the mean ± SEM, *n* = 3. **(F)** Similar gross morphology of the epididymis in WT, Sun5^+/–^ and Sun5^–/–^ mice.

The transcripts of mouse Sun5 are highly conserved in the coding region, so we applied TALEN technology to construct a Sun5^–/–^ mouse model (Figure 1A). The target of the TALEN enzyme was designed to disrupt exon 4 to generate the founder (F0). F0 was mated with WT mice to obtain F1 offspring. F1 generation mice with deletion of two bases (TC) in exon 4 were selected for mating to produce F2 generation mice. We amplified the Sun5 gene segment containing the mutation site from F2 generation genomic DNA using specific primers and sequenced it. Finally, Sun5^–/–^ mice were obtained with TC homologically deficient in exon 4 (Figures 1B,C). Bioinformatics analysis showed that Sun5^–/–^ mice cannot produce intact Sun5 protein.

Then, we confirmed the expression of Sun5 in the testes of WT and Sun5^+/–^ mice but found complete deficiency in Sun5^–/–^ mice by western blot (Figure 1D). All mice survived, and there was no significant difference in the body weights of WT and Sun5^–/–^ mice at 12 weeks postnatal (Figure 1E). In addition, the size and morphology of Sun5^+/–^ and Sun5^–/–^ testes were comparable to those of WT testes by visual inspection (Figure 1F).

### Deficiency of Sun5 in Mice Leads to Acephalic Spermatozoa Syndrome

**FIGURE 2:**
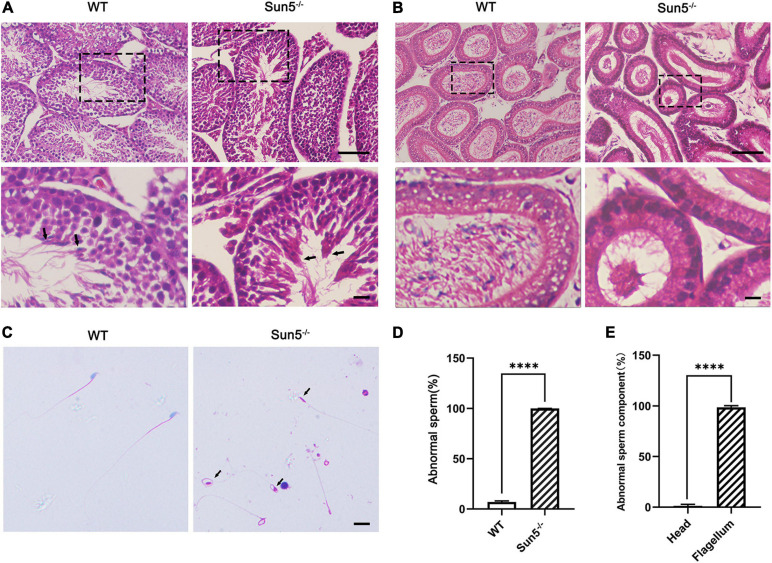
Sun5 deficiency leads to decapitated sperm in Sun5^–/–^ mice. **(A,B)** Histological HE staining of WT and Sun5^–/–^ testes **(A)** and epididymis **(B)**. The arrows indicate the spermatozoa near the lumen of the testis. Bars: upper panel, 100 μm; lower panel, 50 μm. **(C)** HE staining of epididymal sperm smears showing decapitated sperm in Sun5^–/–^ mice. The arrows indicate the remaining cytoplasmic droplets. Bars: 50 μm. **(D,E)** The percentage of abnormal sperm in WT and Sun5^–/–^ mice **(D)** and the percentage of abnormal sperm components in Sun5^–/–^ mice **(E)**. Data are presented as the mean ± SEM, *n* = 3. ^****^P < 0.0001.

**FIGURE 3:**
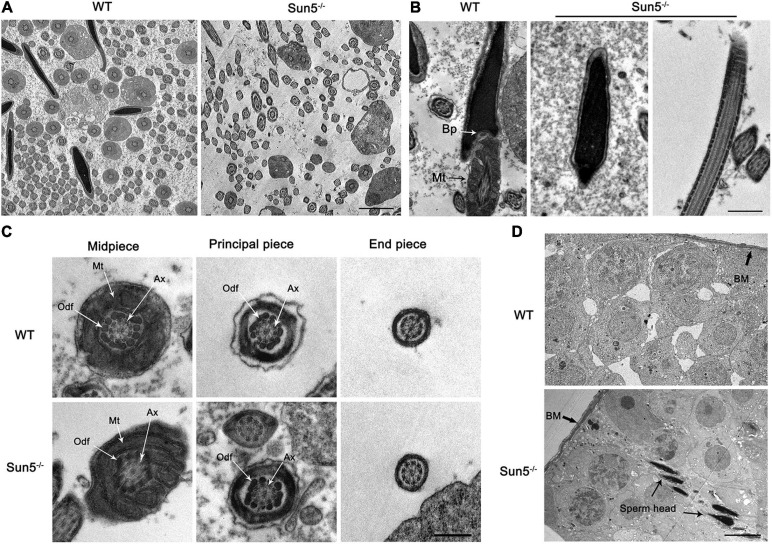
Damaged HTCA of spermatozoa and disordered mitochondria in Sun5^–/–^ mice revealed by TEM. **(A)** Overall perspective of spermatozoa in the epididymal lumen. Bars: 10 μm. **(B)** Longitudinal section of epididymal sperm in WT and Sun5^–/–^ mice. Bp, basal plate; Mt, mitochondria. Bars: 2 μm. **(C)** The cross section of the midpiece, principal piece and end piece of the flagellum. Ax, axoneme; Odf, outer dense fiber. Bars: 200 nm. **(D)** The appearance of the head near the basement membrane (BM). Bars: 10 μm.

To investigate the relationship between Sun5 and acephalic spermatozoa syndrome, we examined the testes and epididymides of WT and Sun5^–/–^ mice histologically. The results of hematoxylin-eosin (HE) staining of the testes did not reveal any severe disruptions in spermatogonia and spermatocytes in the testes of Sun5^–/–^ mice; however, only several (4.20 ± 1.92, *n* = 5) sperm heads were aligned along each lumen in Sun5^–/–^ mice compared with dozens (23.20 ± 4.82, *n* = 5) of sperm heads along each lumen in WT mice (*P* < 0.0001) (Figure 2A). Moreover, the components of the epididymides in Sun5^–/–^ mice were significantly different from those in WT mice. The epididymal lumens of WT mice were full of sperms (30.44 ± 7.30 sperm heads counted, *n* = 9) in each lumen, but the number of sperm heads was significantly decreased (*P* < 0.0001) in Sun5^–/–^ mice (2.44 ± 1.67 sperm heads in each lumen, *n* = 9) (Figure 2B). Further analysis revealed that no healthy spermatozoa were found in Sun5^–/–^ mice (Figures 2C,D). Examination of the Sun5^–/–^ epididymides showed that approximately 98% of the sperm components were headless flagella, and the rest were only sperm heads or sperms with abnormal head-tail connection (Figure 2E). To further identify the potential structural defects, the ultrastructure of the testes and epididymides of WT and Sun5^–/–^ mice was examined by transmission electron microscopy (TEM). We examined 10 fields of 5 μm × 5 μm in the epididymides of four Sun5^–/–^ mice, and only one sperm head was observed. In the WT mice, seven to eight sperm heads could be seen per field of vision on average (Figure 3A). The head and tail of the sperm were tightly connected with clear basal plates and mitochondria neatly arranged along the axoneme in the epididymides of WT mice, whereas the basal plate was not present in spermatozoa in Sun5^–/–^ mice (Figure 3B). Further analysis revealed that the “9 + 2” structure of the axoneme in the sperm tail in Sun5^–/–^ mice seemed to be similar to that in WT mice, but the ODFs and mitochondrial arrangement were disordered in midpiece of the sperm tail (Figure 3C).

About 98% spermatozoa are headless, and the sperm head is barely visible in the epididymides in Sun5^–/–^ mice, suggesting that spermatozoa have lost their heads before entering the epididymides. Thus, we examined the ultrastructure of the testes carefully to determine where the acephalic spermatozoa head was lost in Sun5^–/–^ mice. To our surprise, the sperm heads of Sun5^–/–^ mice were surrounded by Sertoli cells near the basement membrane, suggesting that the decapitated sperm heads were phagocytosed and degraded by Sertoli cells (Figure 3D). Taken together, these results demonstrate that Sun5 deficiency leads to male sterility by producing sperm with severe head-tail breakage with close to 100% deformity.

### Sperm Flagella Were Detached From the Nucleus at Steps 9–10 in Sun5^–/–^ Mice

**FIGURE 4:**
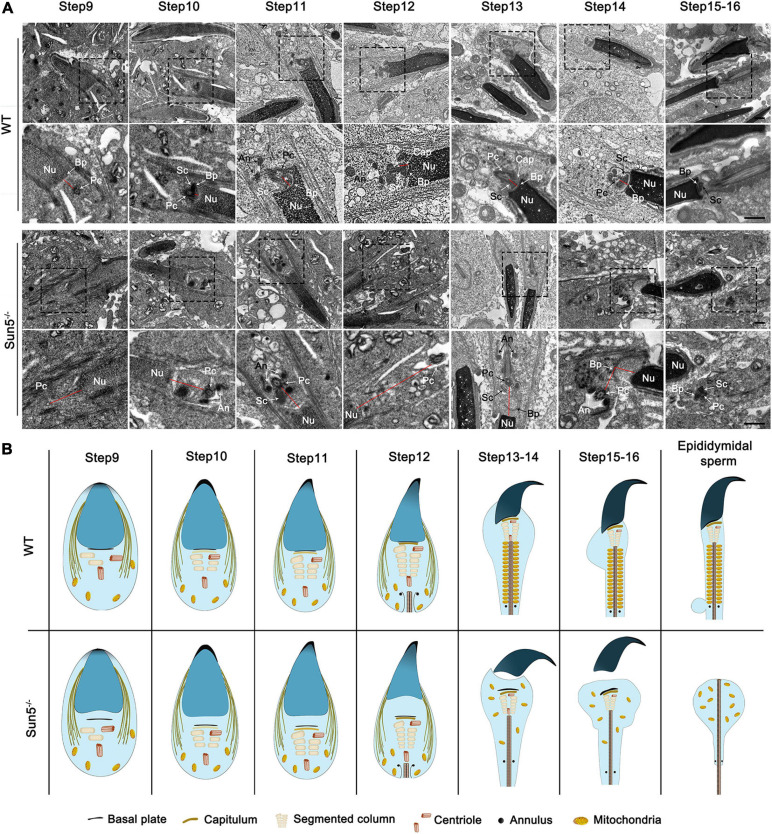
Stepwise development of the centrosome and HTCA in WT and Sun5^–/–^ mice. **(A)** The ultrastructure of steps 9–16 spermatids in WT and Sun5^–/–^ mice by TEM. In step 9 spermatids, the proximal centriole (Pc) appeared at the caudal side of the nucleus (Nu) and gradually assembled the structure of the HTCA. The spacing between the proximal centriole and nucleus increased in Sun5^–/–^ mice compared with WT mice. In subsequent development, the formation of the capitulum (Cap), segmented column (Sc) and annulus (An) was observed in both WT and Sun5^–/–^ mice. However, the HTCA could not attach to the nuclear envelope tightly in Sun5^–/–^ mice. The complete removal of the spermatozoa head from the flagellum occurred during the step 14 manchette disappearance stage. The red line represents the distance between the nuclear membrane and the centrosome. Bars: 2 μm (above) and 1 μm (below). **(B)** Schematic representation of the stepwise development of the centrosome and HTCA in WT and Sun5^–/–^ mice based on TEM.

To investigate the exact time of sperm head and tail fracture, we next focused on the assembly of the HTCA during the differentiation of haploid spermatids. In both WT and Sun5^–/–^ mice, the morphology of round spermatids was similar to each other (Supplementary Figure 2). During spermiogenesis, nucleus began to coagulate and elongate at step 9, with a clear manchette structure. In step 9 spermatids of WT mice, the proximal centriole appeared at the caudal side of the nucleus and gradually assembled the structure of HTCA (Figure 4A). In successive steps of WT, the basal plate-HTCA complex invaginated and attached to the nuclear envelope of the concave implantation fossa (Figure 4A), whereas in Sun5^–/–^ mice, although HTCA could be assembled, the HTCA was detached from the nucleus at steps 9–10. We observed that the space between the centrosome and the nucleus was increased during successive steps 9–13 (Figure 4A). With the disappearance of the manchette at steps 13–14, a visible break in the head-to-tail linkage occurred in the spermatozoa (Figure 4A). At steps 15–16, the head and tail junctions of the spermatids were obviously broken, with the basal plate far away from the implanted fossa (Figure 4A). In summary, TEM analysis of the developmental steps of the spermatozoa confirmed that the absence of Sun5 caused a failure of HTCA for attachment to the nucleus in early developing spermatids, thus leading to the breakage of the head-to-tail connection (Figure 4B).

### Sun5 Interacts With Nesprin3 to Form a Spermiogenesis-Specific LINC Complex and Is Involved in Spermatozoa Head-to-Tail Connection

**FIGURE 5:**
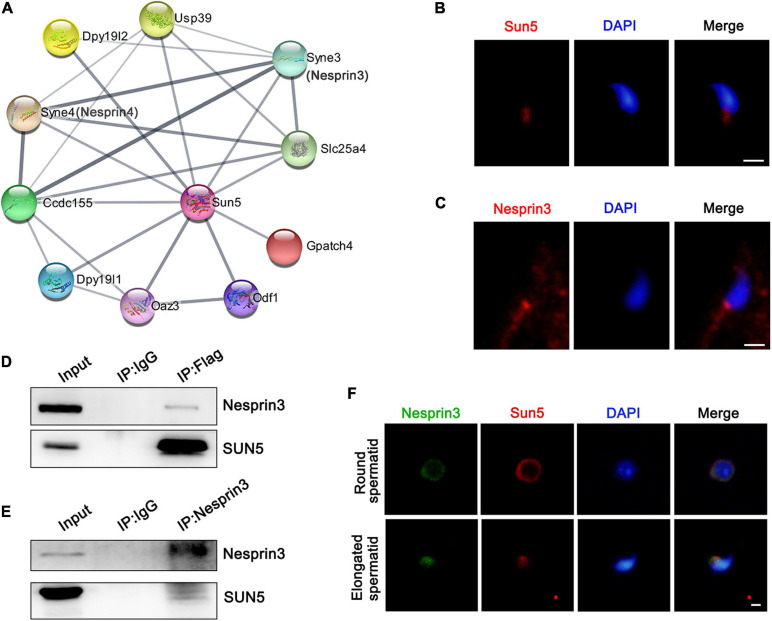
Interaction between SUN5 and Nesprin3 forms the LINC complex. **(A)** Interaction network analysis of SUN5 in mice by the STRING database. **(B)** The localization of SUN5 in spermatozoa by immunofluorescence analysis in mice. Bars: 2.5 μm. **(C)** The localization of Nesprin3 in spermatozoa by immunofluorescence analysis in mice. Bars: 2.5 μm. **(D,E)** The physical interaction between SUN5 and Nesprin3 in vitro validated by co-IP analysis. **(F)** Colocalization of Sun5 with Nesprin3 in different stages of mouse spermatid development by immunofluorescence analysis. Bars: 5 μm.

From the TEM results, we further explored the molecular mechanism by which the flagellum anchors to the nucleus. To gain insights into the molecular function of SUN5, the STRING database was used to analyze the possible interaction partners of Sun5, and a total of 10 potential interacting proteins were identified, such as Nesprin3, Nesprin4, Odf1, Oaz3, Ccdc155, and Slc25a4. We noticed that both Nesprin3 and Nesprin4, belonging to the KASH family, might interact with Sun5 in mice (Figure 5A). Sun5 was located at the coupling apparatus in spermatozoa (Figure 5B). Nesprin3 was located posterior and anterior to the nucleus (Figure 5C). And Sun5 and Nesprin3 shared a similar region at the posterior pole. Different from Nesprin3, Nesprin4 was detected only in the meiosis stage and not in the later haploid sperm development stage (Supplementary Figure 3). Thus, we speculated that Sun5 and Nesprin3 could form the LINC complex involved in spermatozoa head-to-tail connections. Subsequently, we performed a co-IP assay using specific antibodies to confirm the interaction between SUN5 and Nesprin3. In the immunoprecipitants of anti-Flag antibodies, both SUN5 and Nesprin3 were detected in pLVX-IRES-Puro-SUN5-Flag HepG2 cells (Figure 5D). In the immunoprecipitants of anti-Nesprin3 antibodies, both Nesprin3 and SUN5 were also detected (Figure 5E). In addition, the actual interaction between Sun5 and Nesprin3 was confirmed by the co-IP assay in mouse testicular lysates (Supplementary Figure 4). Another piece of evidence supporting their interaction was that Sun5 colocalized with Nesprin3 and gradually approached the connecting piece during spermiogenesis (Figure 5F). These results confirm that SUN5 and Nesprin3 are indeed bona fide interaction partners forming a spermiogenesis-specific LINC complex involved in spermatozoa head-to-tail junctions.

### Sun5 Is Necessary for Nesprin3 Localization in Spermatozoa Development

**FIGURE 6:**
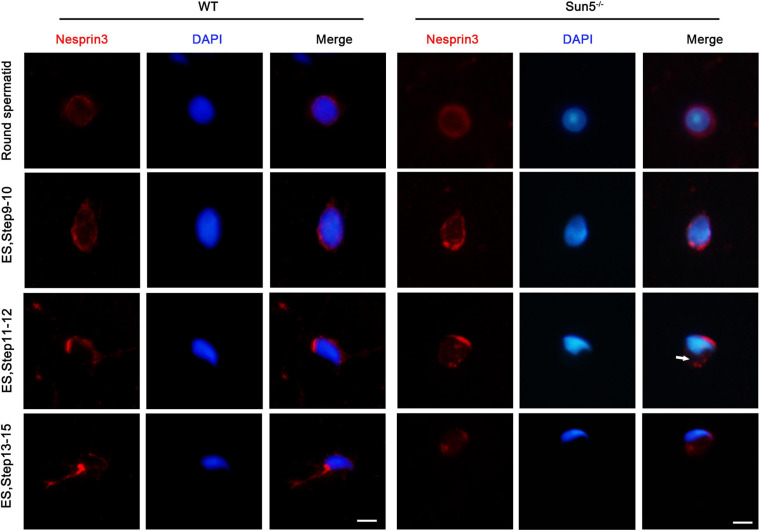
Sun5 deficiency changed the localization of Nesprin3. In round spermatids, Nesprin3 was detected in the nuclear membrane in WT mice but in the perinuclear cytoplasm in Sun5^–/–^ mice. In steps 9–10 and 11–12, Nesprin3 moved anterior and posterior to the nucleus with cell elongation in WT mice, whereas it migrated to the anterior region to a greater degree and no longer clung to the implantation fossa at the posterior region in Sun5^–/–^ mice. In steps 13–15, Nesprin3 was located at the acrosome region and the implantation fossa in WT mice, whereas it lost its localization in the nuclear membrane in Sun5^–/–^ mice. ES, elongated spermatid. Bars: 5 μm.

SUN domain proteins are required for the localization of KASH domain proteins in the nuclear membrane (Kmonickova et al., 2020). To investigate the role of the LINC complex Sun5/Nesprin3 in sperm head and tail connections, we explored the localization of Nesprin3 in the spermatids of Sun5^–/–^ mice. At the round spermatid stage, Nesprin3 was detected in the nuclear membrane in WT mice, while it was detected in the perinuclear cytoplasm in Sun5^–/–^ mice. In steps 9–10 of WT mice, Nesprin3 moved to the anterior and posterior regions of the nucleus along with cell elongation and was finally located at the acrosome region and the implantation fossa (Figure 6). Without the interaction or tractive force by Sun5, Nesprin3 no longer clung to the implantation fossa at the posterior region in elongated spermatids (Figure 6). These results indicated that Sun5 was necessary for correct Nesprin3 localization in spermiogenesis.

### The LINC Complex of Sun5/Nespein3 Regulated Centrosome Docking to the Nucleus

The sperm centrosome has essential functions in the formation of the flagellum and the connection between the head and tail. During spermiogenesis, the distal centriole orients towards the cell membrane to exclusively form the flagellum (Avidor-Reiss et al., 2020). The end of the proximal centriole emanates microtubules to dock to the nuclear membrane, pulling the centrosome to the nucleus, and then a stable connection between the centrosome and nucleus is formed (Wu et al., 2016; Galletta et al., 2020). Previous studies demonstrated that LINC complexes are required for the attachment of the centrosome to the nucleus. SUN5 is located in the inner nuclear membrane, while Nesprin3 is located in the outer nuclear membrane (Kmonickova et al., 2020). Nesprin3 interacts with plectin, MACF and BPAG1 to contact intermediate filaments (IFs) and microtubules to regulate centrosome (Wiche, 1998; Ketema et al., 2007; Cartwright and Karakesisoglou, 2014). Morgan et al. (2011) found that the downregulation of Nesprin3 caused increased spacing between the centrosome and nucleus. Due to the deviated position of Nesprin3 in Sun5^–/–^ mice, we examined the TEM images carefully to determine the position of the centrosome. The results showed that the centrosome was distant from the nuclear envelope starting at step 9 in Sun5^–/–^ mice (Figure 4A). During the development of spermatids, the distance between the nucleus and the centrosome increased significantly (Figure 4A and Supplementary Figure 5). This result indicated that damage to the Sun5/Nesprin3 LINC complex caused the inability of the centrosome to dock to the nuclear envelope, leading to the fracture of the sperm head-to-tail connection during spermatozoa development.

### Dysfunction of Sun5 Changed the Testis Protein Expression Profile

**FIGURE 7:**
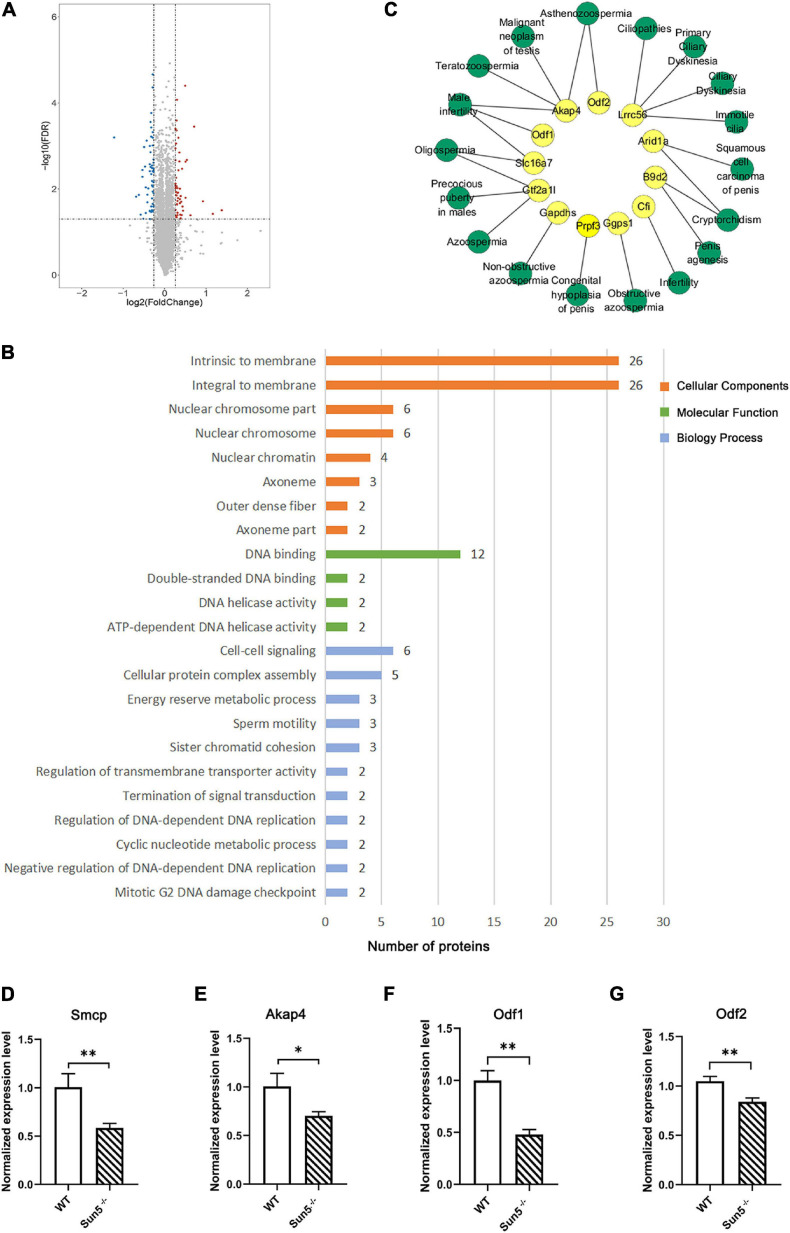
Identification and functional annotation of differentially expressed proteins (DEPs) in testes of WT and Sun5^–/–^ mice. **(A)** Volcano plot of fold change and FDR for 6436 quantified proteins. **(B)** GO enrichment analysis of cellular components, molecular functions and biological processes of DEPs in Sun5^–/–^ mice. **(C)** The network of downregulated proteins and reproductive diseases in humans was annotated by the DisGeNET database. Yellow represents DEPs, and green represents reproductive diseases. **(D–G)** Validation of DEPs related to spermatogenesis at the mRNA level. Data are presented as the mean ± SEM, *n* = 3. ^∗^*P* < 0.05, ^∗∗^*P* < 0.01.

To understand the effect of SUN5 deficiency on the testicular protein expression profile, iTRAQ was performed to analyze testicular protein expression in WT and Sun5^–/–^ mice. A total of 6436 proteins were identified under the 1% FDR (false discovery rate) filtration standard. We screened 103 DEPs containing at least one unique peptide segment with fold change >1.2 and *P*-value < 0.05. The numbers of upregulated and downregulated proteins were 47 and 56, respectively (Figure 7A). To understand the physiological function of the identified differential proteins, these proteins were classified by enrichment analysis. GO enrichment analysis revealed the effects of DEPs on cellular components, molecular functions and biological processes (Figure 7B). Specifically, DEPs were mainly located in the outer dense fibers, membranes and axonemes as part of the cellular component category. In the molecular function category, the DEPs mainly included DNA binding proteins, which we speculated were degraded due to lost interaction with the nucleoskeleton as a result of Sun5 deficiency. In biological processes, these proteins were overrepresented in cell differentiation, cell–cell signaling, cellular protein complex assembly, and sperm motility. We further analyzed the relationship between downregulated proteins and human reproductive diseases by DisGeNET and Cytoscape and found that proteins such as Odf1, Odf1, and Akap4 were involved in asthenozoospermia, teratozoospermia, and male infertility (Figure 7C).

Next, we focused on the downregulated proteins related to reproductive spermatogenesis (Supplementary Table 2) and verified the downregulation of some proteins at the mRNA level, such as Smcp (sperm mitochondrial-associated cysteine-rich protein), Akap4 (A-kinase anchor protein 4), Odf1, and Odf2 (Figures 7D–G), indicating that Sun5 may regulate the expression of some reproductive-related genes *via* the cytoskeleton or other mechanisms. These results indicated that Sun5 deficiency damaged the biological process of spermatogenesis and that these proteins may contribute to the connection of the sperm head and tail.

## Discussion

There is mounting evidence showing that acephalic spermatozoa syndrome is characterized by a specific genetic origin (Zhu et al., 2016; Sha et al., 2018b; Sha et al., 2019). SUN5 mutation is an important cause of this syndrome, which has the highest genetic contribution rate (Zhu et al., 2016; Sha et al., 2018b). In this study, we generated Sun5^–/–^ mice by selecting exon 4 of the Sun5 gene as the disruption target and found that the spermatozoa in Sun5^–/–^ mice were headless. Exon 10 was targeted to knock out the Sun5 gene, and the mice also showed the phenotype of acephalic spermatozoa syndrome (Shang et al., 2017). This confirmed that SUN5 mutation is a causative factor in acephalic spermatozoa syndrome.

To determine the primary cause of damage to spermatozoa in Sun5^–/–^ mice, we analyzed the fine structure of the testis and found that before steps 13–14, the sperm head and tail were connected due to the presence of manchettes, but the HTCA and flagellum were detached from the nucleus in steps 9–10 during spermatid elongation. With the manchette disappearing in steps 13–14, the head and tail appeared to be visibly broken. Further studies revealed that the LINC complex Sun5/Nesprin3 was crucial for the sperm head-to-tail junction.

The LINC complexes, which connect the nucleoskeleton and cytoskeleton, are deemed to play an important role in spermatogenesis (Kmonickova et al., 2020). The Sun1η/Nesprin3 complex is localized anterior and posterior to the nucleus of spermatid during early spermiogenesis. However, as the spermatid elongates, the Sun1η/Nesprin3 complex is excluded from the implantation fossa and is not involved in sperm head-to-tail junctions (Göb et al., 2010). Sun3 and Sun4 interact with Nesprin1 and are colocalized in the manchette, excluding the implantation fossa and playing a critical role in sperm head formation (Pasch et al., 2015). In spermiogenesis, Sun4 is required for tightening of the tail anchorage to the lateral parts of the basal plate, and the head-to-tail linkage is diminished but still present in Sun4-deficient germ cells (Yang et al., 2018). Sun4 does not participate in the centrosomal-nucleus connection, and the position of the centrosome appears to be unaffected after Sun4 deletion (Pasch et al., 2015). Nesprin2 and Nesprin4 were not detected in the postmeiotic stage (Göb et al., 2010). Postmeiotic germ cells also lack Sun2 (Göb et al., 2010), and Sun2 knockout mice are fertile, indicating that Sun2 may not be necessary for spermatogenesis (Lei et al., 2009). Thus, Sun1, Sun2, Sun3, and Sun4 may not be the main factors involved in the sperm head-to-tail junction. Through our experiments, the LINC complex Sun5/Nesprin3 was found to be crucial and unique for sperm head-to-tail junction.

SUN5 is a structural protein located at the head-to-tail junction of sperm. We found that deletion of Sun5 using TALEN technology resulted in altered localization of Nesprin3 in elongated spermatids and increased the distance between centrosome and nucleus. Nesprin3 consists of a KASH domain at the C-terminus and a series of SR domains at the N-terminus. The N-terminus of Nesprin3 could interact with plectin, MACF and BPAG1 and thereby contact IFs and microtubules to regulate centrosome (Wiche, 1998; Ketema et al., 2007; Cartwright and Karakesisoglou, 2014). The centrosome, constituting the microtubule organizing center (MOCT) of cells (Azimzadeh, 2020), is the center of attachment between the sperm head and tail during the formation of flagella (Galletta et al., 2020). During spermiogenesis, the proximal centriole, associated with the nucleus, inserts into the implantation fossa, which is opposite to the acrosome, and the segmented columns and the capitulum together form a connecting piece in the region, while the distal centriole sets the template for the flagellum (Fawcett and Phillips, 1969; Fawcett, 1975). Thus, it is a prerequisite for the tight connection between the head and tail of spermatozoa that the centrosome docks into the nuclear membrane during early spermiogenesis (Chemes and Alvarez, 2012). Studies showed that knockdown of Nesprin3 increased the spacing between the nucleus and the centrosome, indicating that Nesprin3 was crucial for the correct position of the centrosome and the attachment of the centrosome to the nuclear envelope (Morgan et al., 2011). Based on these reasons, we speculate that during spermiogenesis, Nesprin3 associates with microtubules and pulls the centrosome to the nucleus. When the Sun5/Nesprin3 LINC complex was damaged, the centrosome became distant from the nucleus, leading to failure to anchor the HTCA to the implantation fossa, thus resulting in rupture of the sperm head-to-tail connection.

In addition to changing the localization of Nesprin3, the deletion of Sun5 also affected the expression of a whole set of proteins. We found that many proteins involved in spermatogenesis were downregulated after Sun5 knockout and the mRNA expression of Smcp, Akap4, Odf1, and Odf2 was also downregulated, indicating that the function of Sun5 is important for spermatogenesis-related gene expression. Similarly, the downregulation of Odf1 was also detected in the sperm of acephalic spermatozoa syndrome patients with SUN5 mutations (Sha et al., 2018b). Odf1 is located at the manchette, HTCA and flagellum (Yang et al., 2012; Tapia and Hoyer-Fender, 2019). The downregulation of Odf1 in Odf1^+/–^ mice causes relaxation between the nuclear membrane and the capitulum of spermatozoa (Yang et al., 2014). Homozygous Odf1^–/–^ males were infertile due to decapitated sperm in the epididymis (Yang et al., 2012, 2014). These results suggest that a series of downregulated proteins induced by Sun5 deficiency may be involved in sperm head-to-tail connections, such as the Odf1, a crucial sperm neck protein.

**FIGURE 8:**
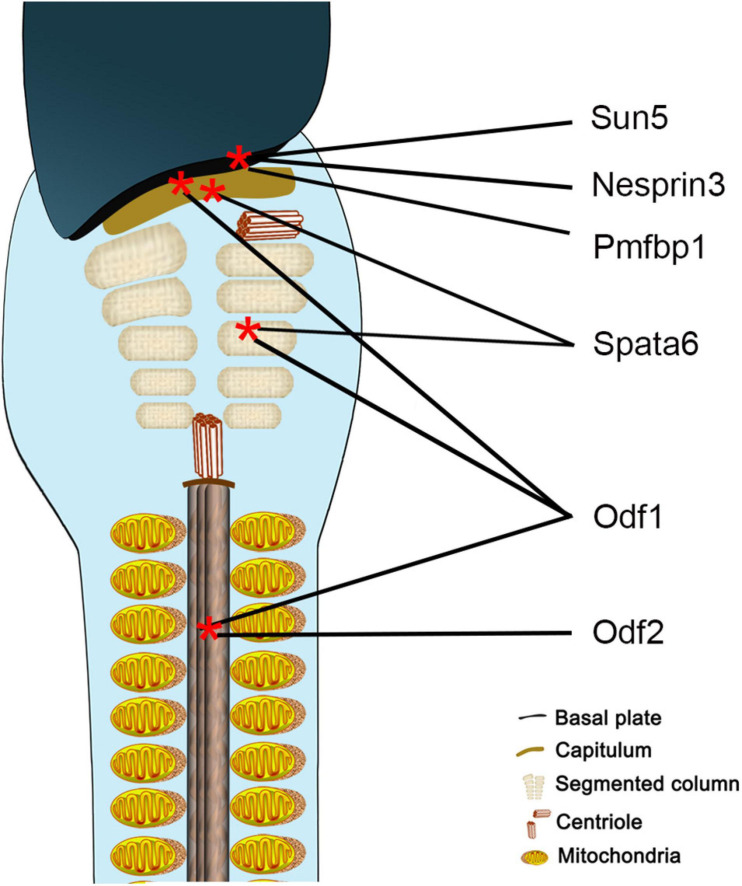
Schematic representation of localization of proteins involved in sperm head-to-tail connection. Sun5, Nesprin3, and Pmfbp1 are located at the implantation fossa, Spata6 at the capitulum and segmented column, Odf1 at the HTCA and flagellum, and Odf2 at the flagellum.

Sun5 acts synergistically with a variety of proteins to tether the flagellum tightly to the implantation fossa, but the component is complex and unclear. Researchers found that Sun5 cooperated with Pmfbp1 and Spata6 to form a sandwich-like structure in the HTCA, with Sun5 at the top of the structure and Spata6 at the bottom (Figure 8), but they could not interact with each other directly (Zhu et al., 2018). Thus, the connection complex tethering the flagellum to the nucleus remains unclear. In our present study, the interaction between Sun5 and Nesprin3 provided new insight into the composition of the complexes (Figure 8). This unique LINC complex in the sperm neck, associating with the microtubules, mediates the anchoring of centrosome and caudal structures to the implantation fossa.

In addition, we used TALEN technology to generate the Sun5 knockout model by engineering exon 4 of the Sun5 gene. During the breeding process, each generation of mice was genotyped and sequenced. So far, we have not identified any off-target site or off-target phenotype.

Taken together, our work confirms the crucial role of the LINC complex Sun5/Nesprin3 in the sperm head-to-tail junction and provides new mechanistic insights into the role of Sun5 in sperm head-to-tail connections during spermatogenesis. Further research on the molecular mechanism by which Sun5 mediates sperm head-to-tail connections and gene therapy in acephalic spermatozoa with Sun5 mutations is needed, which is of significance to understand the pathogenesis and clinical treatment of acephalic spermatozoa syndrome.

## Data Availability Statement

The datasets presented in this study can be found in online repositories. The names of the repository/repositories and accession number(s) can be found in the article/ Supplementary Material.

## Ethics Statement

The animal study was reviewed and approved by the Experimental Animals Ethics Committee of The Third Xiangya Hospital, Central South University.

## Author Contributions

XX designed the study. YZ, LY, XZ, and XN performed the study. YZ, LH, and GL analyzed the data. YZ wrote the first draft of the manuscript. LH and GL discussed the results and edited the manuscript. All authors have approved the final manuscript.

## Conflict of Interest

The authors declare that the research was conducted in the absence of any commercial or financial relationships that could be construed as a potential conflict of interest.

## Footnotes

1 www.ncbi.nlm.nih.gov

2 https://www.string-db.org

3 http://www.disgenet.org

## Supplementary Material

Supplementary Figure 1 Structure of Nesprin1–3. Nesprin3 consists of a C-terminal KASH domain and a series of SRs. Different from Nesprin1 and Nesprin2, Nesprin3 is lack of ABD.

Supplementary Figure 2 TEM analyses of round spermatids in WT and Sun5^–/–^ mice. Bars: 5 μm.

Supplementary Figure 3 The localization of Nesprin4 in germ cells.

Supplementary Figure 4 Co-IP assays validating the physical interactions between Sun5 and Nesprin3 in testis of WT mice. The ratios of gray values of Input, IP by Sun5 or Nesprin3 antibodies and IP by IgG are following: IP by Sun5 antibody detected by Nesprin3, 0.23:1.00:0.00; IP by Sun5 antibody detected by Sun5, 0.32:1.00:0.02; IP by Nesprin3 antibody detected by Nesprin3, 0.37:1.00:0.00; IP by Nesprin3 detected by Sun5, 0.25:1.00:0.04.

Supplementary Figure 5 The distance between centrosome and nucleus. Data are presented as the mean ± SEM, *n* = 5. ^∗∗∗^*P* < 0.001.

Supplementary Table 1 List of primers used for genotyping and validation of DEPs using qRT-PCR.

Supplementary Table 2 Downregulated proteins related to reproductive spermatogenesis and their function.

Supplementary Table 3 Antibody used in this study.
