# Supplementary figures and images for: SUN5 Interacting With Nesprin3 Plays an Essential Role in Sperm Head-to-Tail Linkage: Research on Sun5 Gene Knockout Mice
Source: Front Cell Dev Biol. 2021 Jun 29;9:684826. doi: 10.3389/fcell.2021.684826 (PMC8276135; doi:10.3389/fcell.2021.684826)

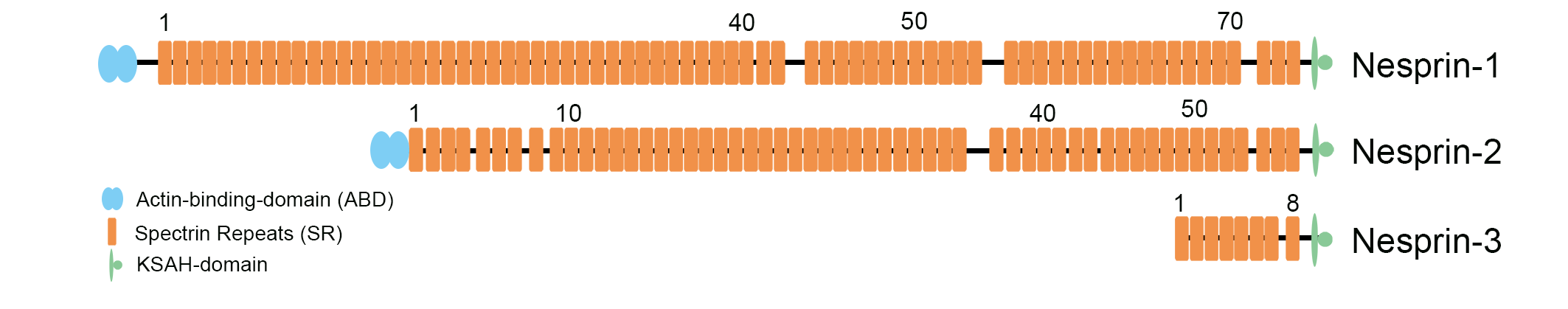

Supplement: Supplementary Figure 1 — Structure of Nesprin1–3. Nesprin3 consists of a C-terminal KASH domain and a series of SRs. Different from Nesprin1 and Nesprin2, Nesprin3 is lack of ABD. [file Image_1.TIF]

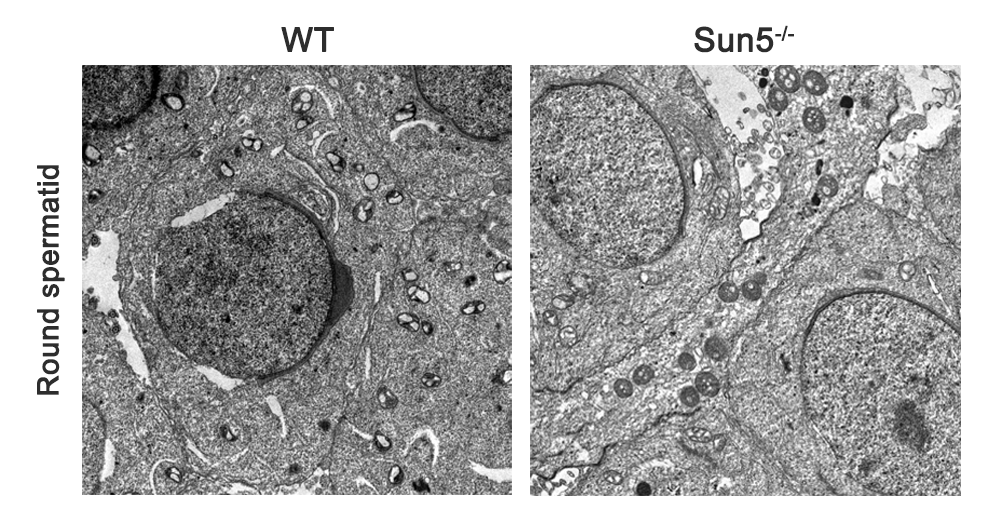

Supplement: Supplementary Figure 2 — TEM analyses of round spermatids in WT and Sun5–/– mice. Bars: 5 μm. [file Image_2.TIF]

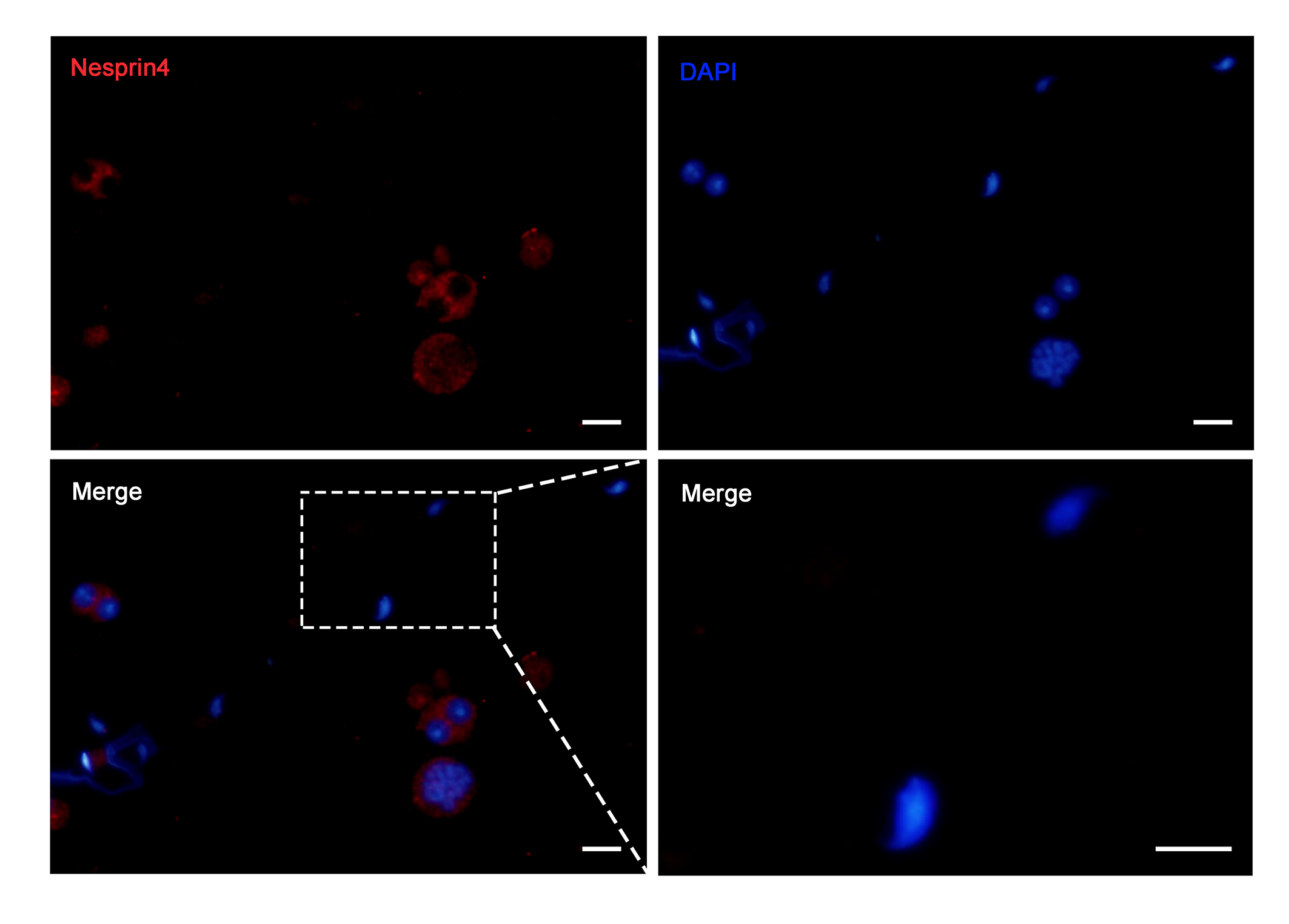

Supplement: Supplementary Figure 3 — The localization of Nesprin4 in germ cells. [file Image_3.TIF]

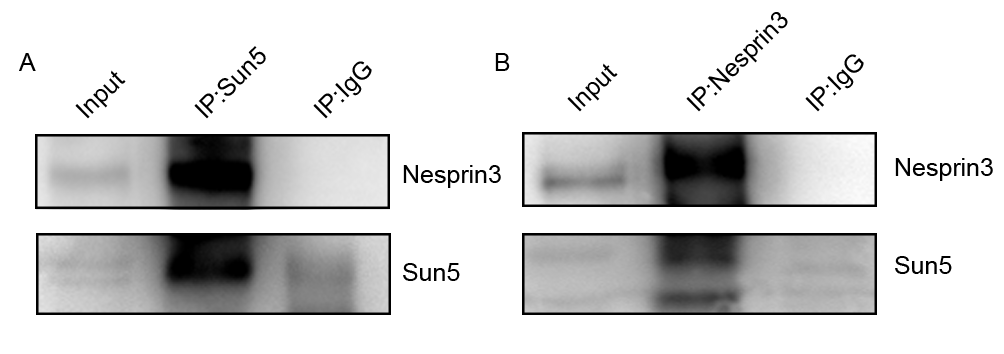

Supplement: Supplementary Figure 4 — Co-IP assays validating the physical interactions between Sun5 and Nesprin3 in testis of WT mice. The ratios of gray values of Input, IP by Sun5 or Nesprin3 antibodies and IP by IgG are following: IP by Sun5 antibody detected by Nesprin3, 0.23:1.00:0.00; IP by Sun5 antibody detected by Sun5, 0.32:1.00:0.02; IP by Nesprin3 antibody detected by Nesprin3, 0.37:1.00:0.00; IP by Nesprin3 detected by Sun5, 0.25:1.00:0.04. [file Image_4.TIF]

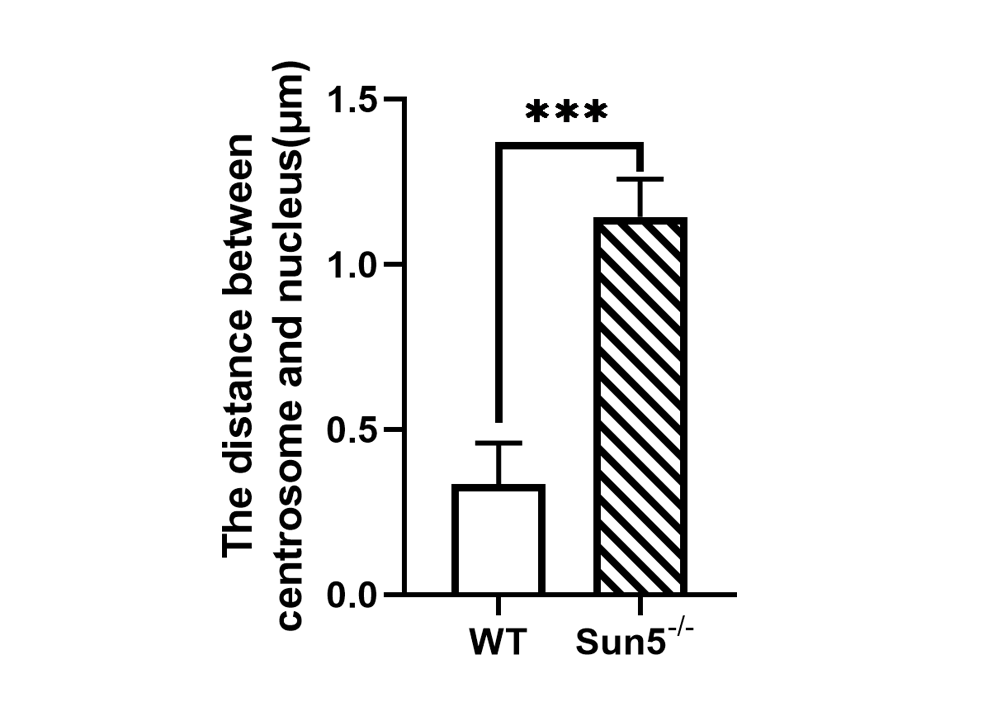

Supplement: Supplementary Figure 5 — The distance between centrosome and nucleus. Data are presented as the mean ± SEM, n = 5. ∗∗∗P < 0.001. [file Image_5.TIF]
