# Supplementary material for: SUN5 Interacting With Nesprin3 Plays an Essential Role in Sperm Head-to-Tail Linkage: Research on Sun5 Gene Knockout Mice
Source: Front Cell Dev Biol. 2021 Jun 29;9:684826. doi: 10.3389/fcell.2021.684826 (PMC8276135; doi:10.3389/fcell.2021.684826)
Supplement: Supplementary Table 1 — List of primers used for genotyping and validation of DEPs using qRT-PCR. [file Table_1.DOCX]

**Supplementary Table 1:**

List of primers used for genotyping and validation of DEPs using qRT-PCR

| **Primer Name** | **Primer Sequence 5’---3’** |
| --- | --- |
| Sun5 check F | CCCAGTGTCCAGGGATGACATTAA |
| Sun5 check R | TGGGTCCACAGAAGGAAGGCA |
| Smcp F | GACTCACTAGACTGCTGAGGA |
| Smcp R | CAACATGGTTTTGGTGGGCA |
| Akap4 F | GTGTCCTCAACTGGCTCCTC |
| Akap4 R | GTGTTTTTGGATGCCGCCAT |
| Odf1 F | GCCCATCGCTCCGCAGTTTA |
| Odf1 R | ATCAGGTTCAAAGCCGCACA |
| Odf2 F | CTCCCCCAGGTTTCCATCG |
| Odf2 R | CCCCTTTCATTCCGCGCTTAT |
| Gapdh F | AGGTCGGTGTGAACGGATTTG |
| Gapdh R | TGTAGACCATGTAGTTGAGGTCA |
