# Supplementary material for: SUN5 Interacting With Nesprin3 Plays an Essential Role in Sperm Head-to-Tail Linkage: Research on Sun5 Gene Knockout Mice
Source: Front Cell Dev Biol. 2021 Jun 29;9:684826. doi: 10.3389/fcell.2021.684826 (PMC8276135; doi:10.3389/fcell.2021.684826)
Supplement: Supplementary Table 2 — Downregulated proteins related to reproductive spermatogenesis and their function. [file Table_2.DOCX]

**Supplementary Table 2:**

Downregulated proteins related to reproductive spermatogenesis and their function

| **Accession** | **Protein name** | **Gene name** | **Description** | **Function** |
| --- | --- | --- | --- | --- |
| P15265 | MCSP | Smcp | Sperm mitochondrial-associated cysteine-rich protein | Involved in sperm motility. Its absence is associated with genetic background dependent male infertility. |
| Q60662 | AKAP4 | Akap4 | A-kinase anchor protein 4 | Major structural component of sperm fibrous sheath. Plays a role in sperm motility. |
| Q9DAM3 | HSPB9 | Hspb9 | Heat shock protein beta-9 | Specific high expressed in the spermatogenic cells from late pachytene spermatocyte stage till elongate spermatid stage. |
| A3KGV1 | ODFP2 | Odf2 | Outer dense fiber protein 2 | A major component of sperm tail outer dense fibers (ODF). Functions as a general scaffold protein that is specifically localized at the distal/subdistal appendages of mother centrioles. |
| O70451 | MOT2 | Slc16a7 | Monocarboxylate transporter 2 | Proton-coupled monocarboxylate transporter in mice. |
| Q8C633 | CABS1 | Cabs1 | Calcium-binding and spermatid-specific protein 1 | Detected in mitochondria of step 17 to 18 spermatids. Associated with the mitochondrial inner membrane in step 18 spermatids. |
| Q8R4I4 | TF2AY | Gtf2a1l | TFIIA-alpha and beta-like factor | May function as a testis specific transcription factor. |
| Q5SZT7 | NKAPL | Nkapl | NKAP-like protein | Transcriptional repressor of Notch-mediated signaling. Required for spermatogenesis. |
| Q64467 | G3PT | Gapdhs | Glyceraldehyde-3-phosphate dehydrogenase, testis-specific | Required for sperm motility and male fertility. |
| Q61999 | ODFP1 | Odf1 | Outer dense fiber protein 1 | Component of the outer dense fibers (ODF) of spermatozoa. Functions in maintaining the passive elastic structures and elastic recoil of the sperm tail. |
