# Supplementary material for: SUN5 Interacting With Nesprin3 Plays an Essential Role in Sperm Head-to-Tail Linkage: Research on Sun5 Gene Knockout Mice
Source: Front Cell Dev Biol. 2021 Jun 29;9:684826. doi: 10.3389/fcell.2021.684826 (PMC8276135; doi:10.3389/fcell.2021.684826)
Supplement: Supplementary Table 3 — Antibody used in this study. [file Table_3.DOCX]

**Supplementary Table 3:**

| Antibody used in this study | | | | |
| --- | --- | --- | --- | --- |
| Antibodies | Company | Products | Dilution fold values | Function |
| Rabbit Sun5 Polyclonal antibody | Proteintech | 17495-1-AP | 1:1000,1:100 | Western blotting, Immunostaining |
| Mouse GAPDH Monoclonal antibody | Proteintech | 60004-1-Ig | 1:1000 | Western blotting |
| Rabbit Nesprin3 Polyclonal antibody | Proteintech | 27132-1-AP | 1:100 | Immunostaining |
| Rabbit polyclonal to Nesprin3 | Abcam | ab190010 | 1:1000,1:50 | Western blotting, Co-IP |
| Mouse monoclonal to Nesprin3 | Abcam | ab123031 | 1:100 | Immunostaining |
| Rabbit IgG | Beyotime | A7016 | 1:50 | Co-IP |
| FLAG Tag Rabbit Polyclonal Antibody | Beyotime | AF0036 | 1:50 | Co-IP |
| Cy3-labeled Goat Anti-Rabbit IgG (H+L) | Beyotime | A0516 | 1:200 | Immunostaining |
| FITC-labeled Goat Anti-Mouse IgG (H+L) | Beyotime | A0568 | 1:100 | Immunostaining |
| HRP-labeled Goat Anti-Rabbit IgG(H+L) | Beyotime | A0208 | 1:1000 | Western blotting |
| HRP-labeled Goat Anti-Mouse IgG(H+L) | Beyotime | A0216 | 1:1000 | Western blotting |
